# Surface-Enhanced Raman Spectroscopy Substrates: Plasmonic Metals to Graphene

**DOI:** 10.3389/fchem.2022.832282

**Published:** 2022-03-09

**Authors:** Nikiwe Mhlanga, Thabang A. Ntho, Hleko Chauke, Lucky Sikhwivhilu

**Affiliations:** ^1^ DSI/Mintek Nanotechnology Innovation Centre, Randburg, South Africa; ^2^ Advanced Materials Division, Mintek, Randburg, South Africa; ^3^ Department of Chemistry, University of Witwatersrand, Johannesburg, South Africa

**Keywords:** surface-enhanced Raman spectroscopy (SERS), graphene-enhanced Raman spectroscopy (GERS), electromagnetic, charge transfer, plasmonic

## Abstract

Surface-enhanced Raman spectroscopy (SERS), a marvel that uses surfaces to enhance conventional Raman signals, is proposed for a myriad of applications, such as diagnosis of diseases, pollutants, and many more. The substrates determine the SERS enhancement, and plasmonic metallic nanoparticles such as Au, Ag, and Cu have dominated the field. However, the last decades have failed to translate SERS prototypes into real-life applications. Irreproducibility on the SERS signal that stems from the roughened SERS substrates is the main causative factor for this observation. To mitigate irreproducibility several two-dimensional (2-D) substrates have been sought for use as possible alternatives. Application of 2-D graphene substrates in Raman renders graphene-enhanced Raman spectroscopy (GERS). This account used density functional theory (DFT) substantiated with experimental Raman to compare the enhancement capabilities of plasmonic Au nanoparticles (SERS), graphene substrate (GERS), and coupling of the two SERS and GERS substrates. The DFT also enabled the study of the SERS and GERS systems molecular orbital to gain insight into their mechanisms. The amalgamation of the SERS and GERS occurrence, i.e., graphene doped with plasmonic metallic substrates showed a pronounced enhancement and matched the Au-driven enhancement emanating from both electromagnetic and charge transfer SERS and GERS mechanisms.

## Introduction

Surface-enhanced Raman spectroscopy (SERS) has been intensely studied in the last four decades in analytical chemistry and food safety (Nguyen et al., 2014). The application of SERS in myriad applications is driven by its fingerprinting specificity, multiplexing potential, single-molecule level sensitivity, bioimaging, and bioanalysis capabilities (Miao et al., 2019). SERS is the scientific art of the amplification of a traditional low Raman signal using coinage of roughened metal surface (Ling et al., 2015). Inherently, SERS relies on the SERS substrate for an enhanced signal. It has been studied for the detection of several analytes in diagnostic, food, environmental sectors, and many more. The SERS substrate, as the impetus for this phenomenon, has been explored to a great extent, and conventional roughened metallic nanoparticles (NPs), such as Au, Ag, and Cu, are the cornerstones in SERS applications. Typical noble metal-based SERS substrate entails deposition of the metals on some support, and for the longest time, the support has been solid glass/silicon wafer (Nguyen et al., 2014; Miao et al., 2019). The NPs have been favorable substrates due to their compatible size and richness in localized surface plasmon resonance (LSPR) properties (Miao et al., 2019). However, with SERS prototypes dominating the scientific publication in the last four decades, their translation to real-life applications is a bottleneck (Nguyen et al., 2014).

Envisioned translation of the prototypes to end-user products is hindered by several factors: reproducibility, reliability, poor biocompatibility, stability and flexibility (Miao et al., 2019), cost, environmental safety (Almohammed et al., 2019), poor size and shape control of the NPs (Kim et al., 2014), quantification, and detailed SERS mechanism (Ling et al., 2015). Irreproducibility emanates from random adsorption of target molecules on the roughened metallic substrates (Miao et al., 2019). To mitigate and facilitate ingress of SERS prototypes into everyday use, substrates have expanded into other materials, such as 2-D graphitic substrates.

Application of graphene and its variants in SERS is merited by its flexibility, lightweight, photocatalytic, (Nguyen et al., 2014), uniform Raman signal (Miao et al., 2019), stability, low bulk cost production, biocompatibility (e.g., graphene oxide) (Almohammed et al., 2019), electrical, optical, thermal conductivity, and mechanical applications, molecular orientation and edge defect contributing to charge transfer (Kim et al., 2014), and flatness (Ling et al., 2015). Its flatness yields better manipulation and configuration of molecular distribution (Ling et al., 2015). The application of graphene in Raman is termed graphene-enhanced Raman spectroscopy (GERS) (Ling et al., 2015). Although graphene offers enthralling properties, when compared with the roughened metallic irreproducible signal substrates, it has a lower SERS signal (Almohammed et al., 2019). Mitigation measures to enhance its signals include invoking an externally applied field to aid the charge transfer (Almohammed et al., 2019) and doping with the metals (Miao et al., 2019). Almohammed et al. (Almohammed et al., 2019) studied the GERS electric field hypothesis. They proved that SERS enhancement is enabled by an electric field on aligned semiconducting peptide nanotube–graphene oxide composite structures. The electric field-enhanced charge transfer enabled the detection of glucose and nucleobases with a 10-fold enhancement compared with noble metal SERS. Nitrogen-doped graphene quantum wrapped with Au NPs was investigated as an SERS substrate for detection and cellular imaging. Long stability, biocompatibility, and increased enhancement were observed for the substrate (Miao et al., 2019). The SERS enhancement was driven by both the chemical (CM) and electromagnetic (EM) SERS mechanisms (Miao et al., 2019).

The alluded EM and CM are the adopted SERS mechanisms. EM is governed by the excitation of LSPR that yields a localized electromagnetic field, and CM is the partially resonant charge transfer between molecules and substrate and nonresonate chemical interaction between their ground state (Kim et al., 2014; Miao et al., 2019). Charge transfer is dependent on the Fermi level alignment and internal field (Almohammed et al., 2019). In GERS, EM is promoted by the π−π interactions evoked between graphene substrate and absorbed molecules, which shortens the distance between analyte and substrate and, hence, promotes hot spots, while CM is the charge transfer between the analyte and graphene (Miao et al., 2019).

This paper studies the various SERS substrates, from the traditional roughened metals of Au NPs to the 2-D graphene. It reports a density functional theory (DFT) study corroborated with experimental Raman to understand the SERS enhancement mechanism.

## Computational details

An Orca (Neese, 2012) with a libnt2 library (Valeev, 2021) and built-in libXe version 5.1.0 (Lehtola et al., 2018) was used in this account to investigate different SERS surfaces, from the traditional roughened metallic Au to 2-D graphene. SERS and GERS substrates, Au3, and graphene (19 benzene rings) enhancement capabilities were calculated in the presence of 4-MBA SERS tag using DFT. Optimization and Raman frequency calculations used a BP86 DFT engine coupled with basis sets, def2-SVP (Weigend and Ahlrichs, 2005) for 4-MBA atoms and graphene and def2-TZVP (Weigend and Ahlrichs, 2005) for the Au atoms with effective core potentials (Def2-ECPs) (Dolg et al., 1989a; Dolg et al., 1989b; Andrae et al., 1990; Kaupp et al., 1991; Leininger et al., 1996; Metz et al., 2000; Cao and Dolg, 2001; Peterson et al., 2003). An auxiliary basis: def2/J (Weigend, 2006) was also used to speed up the calculation. Atom-pairwise dispersion correction was achieved with the Becke–Johnson damping scheme (D3BJ) (Grimme et al., 2010; Grimme et al., 2011). Enabled by the use of the conductor-like polarizable continuum (CPCM) model, water solvent was also incorporated in the simulations. The study utilized the subsequent visualization tools: Janpa (Nikolaienko et al., 2014; Yu, 2019) for natural population analysis (NPA), Ibo View (Knizia, 2015) for visualization of intrinsic bond orbitals, and Avogadro (Hanwell et al., 2012) for frequency vibrations visualization.

## Experimental details

MBA (0.02 mM in ethanol) was dropcoated on SERS substrates: Au NPs (the synthesis is detailed in Mhlanga et al., 2019), GERS substrate graphene (Sigma-Aldrich), and on a combination of the SERS and GERS substrates. All the reagents were purchased from Sigma-Aldrich, South Africa. The samples on Raman holders were air dried, and Sketch 1 depicts the sample preparation. The samples were analyzed using a Perkin Elmer station 100 Raman spectroscopy with the following specifications: *λ* = 785 nm, focal length = 750 μm, laser spot area = 2,540 μm, 60% laser power, 3,000–1,000 cm^−1^ range, 20 accumulations, and 5-s exposure time.

## Results and discussion

### The effect of plasmonic and two-dimensional graphene on the enhancement of the Raman signal

**FIGURE 1:**
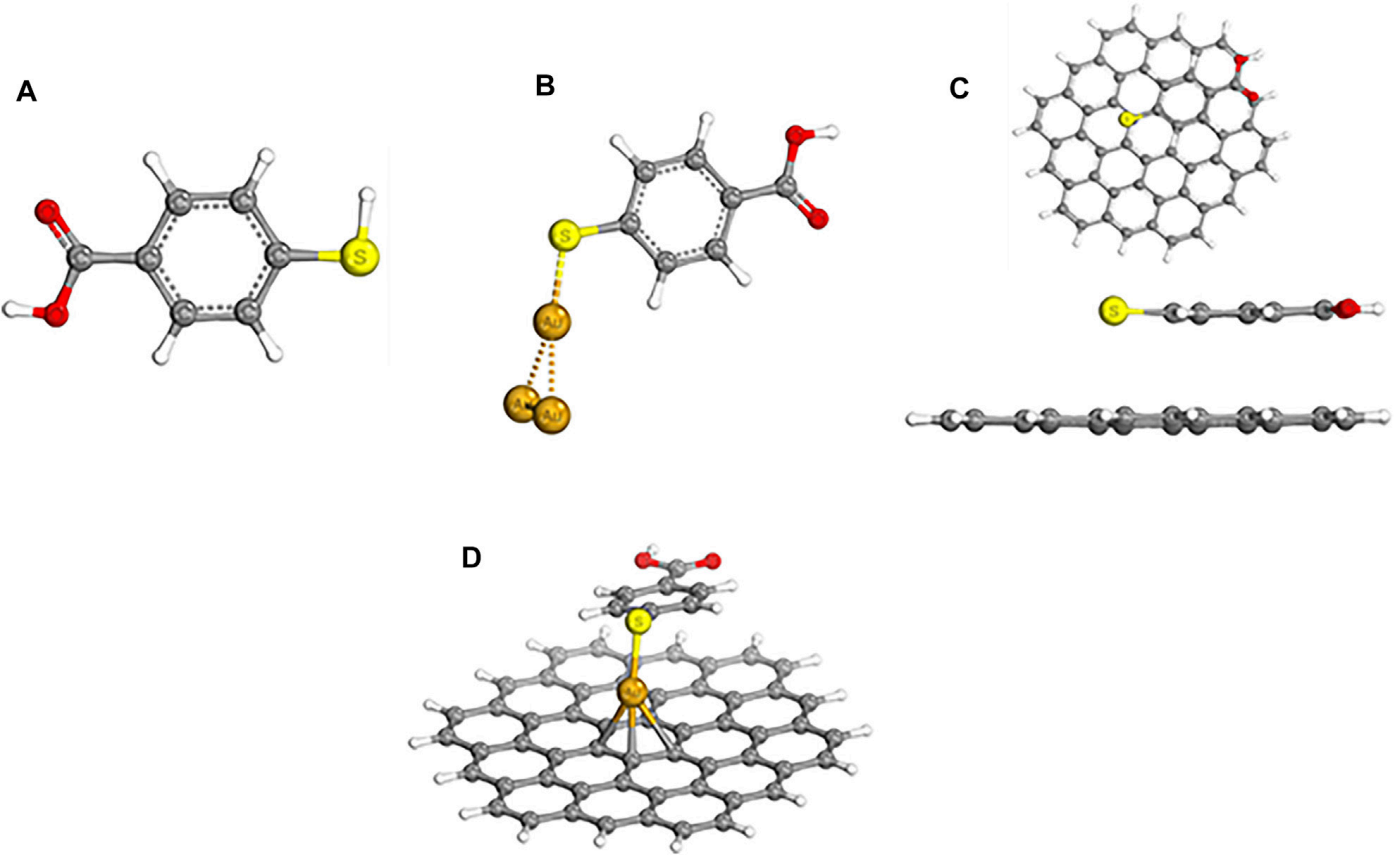
BP86 D3BJ def2-SVP/TZVP def2/optimized chemical structures of **(A)** 4-MBA, **(B)** Au/MBA (SERS), **(C)** top and side views of graphene/MBA [graphene-enhanced Raman spectroscopy (GERS)] and **(D)** Au/GERS (graphene/Au/MBA).

The roughened plasmonic metallic amplification of the Raman signal is reliant on the metallic surface and, hence, termed SERS, while the 2-D graphene-dependent enhancement is GERS. Both SERS and GERS proffers desired amplification of the traditional Raman signal and are used in various applications. The DFT was used as a vehicle to study the SERS and GERS substrates at BP86 D3BJ def2-SVP or TZVP def2/j level of theory, and Figure 1 depicts ground state geometries of the clusters. The same level of theory in a solvent phase (water) was used to calculate the SERS and GERS spectra, and the results corroborated with that of unconjugated 4-MBA (Figure 1A). Chemisorption is observed between the Au–S bond in Figure 1B, and Au–S and Au–C of graphene in Figure 1D with bond lengths of 2.29, 2.29, and 2.32 Å, respectively. The 4-MBA SERS tag is chemisorbed on the substrate with angles of 107.24°C (Figure 1D) and 95.49°C (Figure 1D). Figure 1C shows the bond formation between graphene and 4-MBA, and 4-MBA on Au/GERS inherited a flat conformation.

**FIGURE 2:**
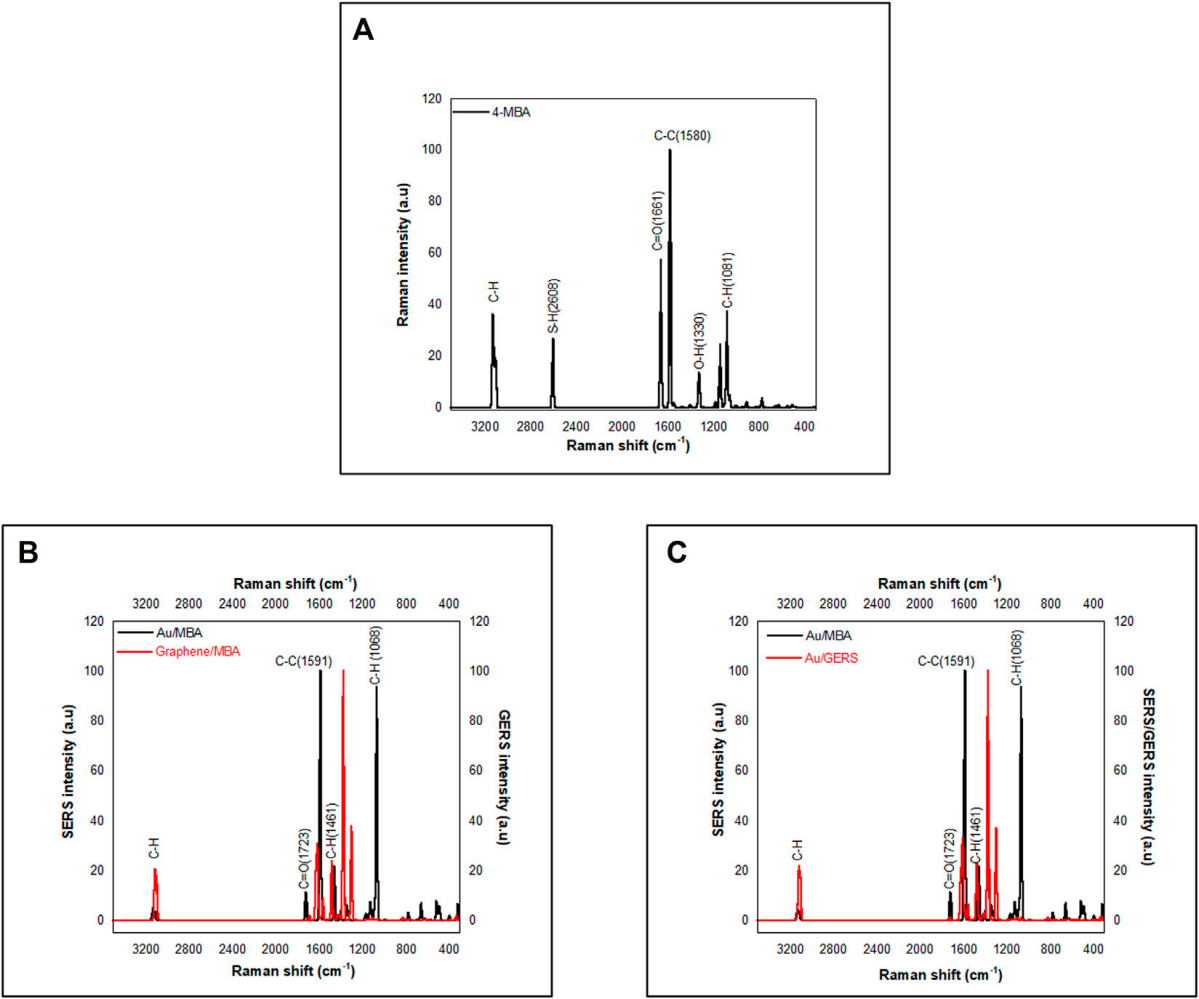
Simulated data. **(A)** 4-MBA. **(B)** Graphene/MBA compared with Au/MBA and **(C)** Au/MBA compared with Au/GERS.

**FIGURE 3:**
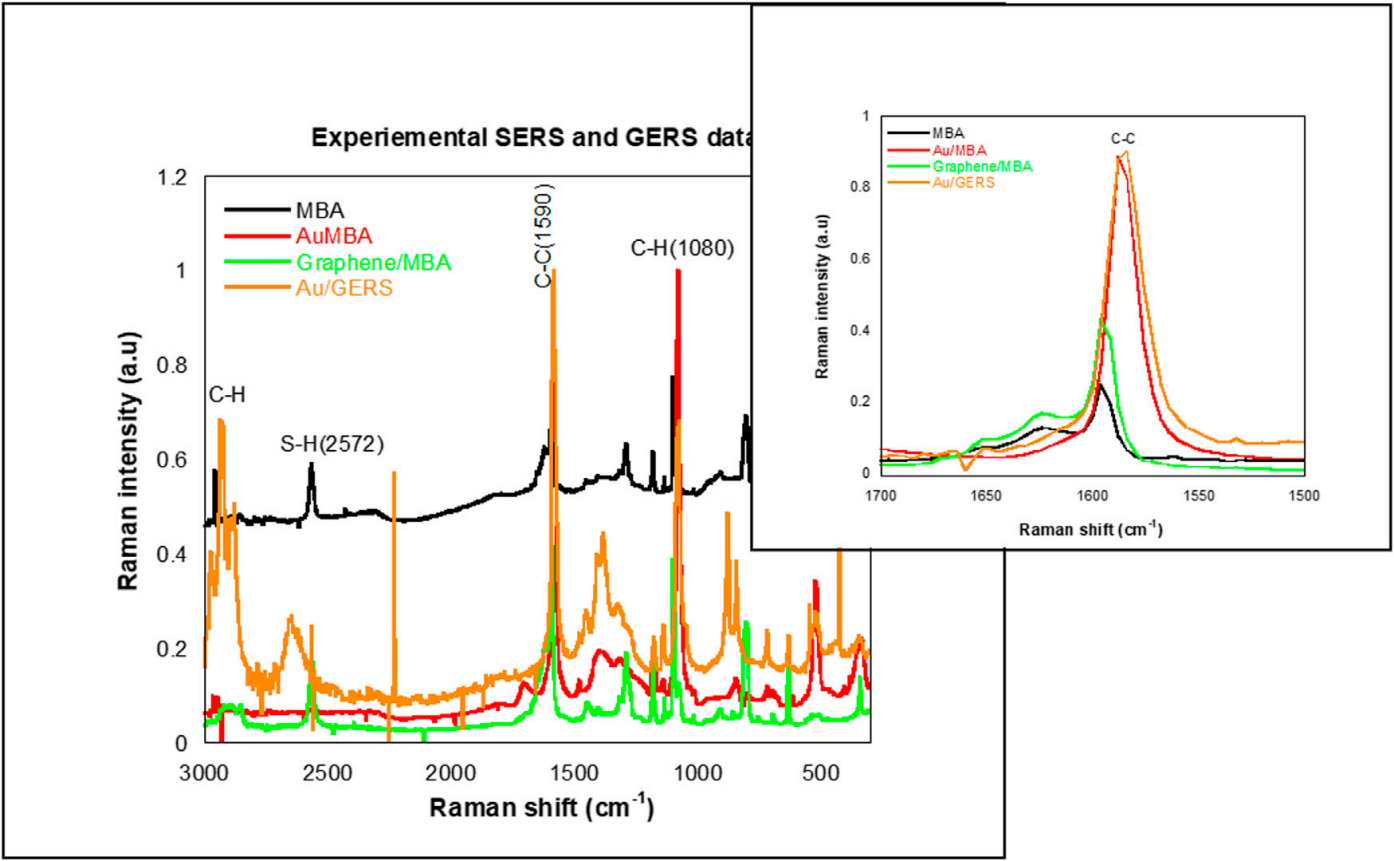
Experimental surface-enhanced Raman spectroscopy (SERS) and GERS data compare MBA, Au/MBA, graphene/MBA, and Au/GERS.

Enhancement capabilities of the three systems, i.e., Au/MBA SERS, GERS graphene/MBA, and SERS + GERS Au/GERS substrates were evaluated *via* frequency calculations, and Raman spectra are shown in Figure 2. A pristine 4-MBA control is shown in Figure 2A, and this account focuses on the labeled peaks: $\nu_{s}$ S-H (2,608 cm^−1^), $\nu_{s}$ C=O (1,661 cm^−1^), $\delta_{s}$ C–C (1,600 cm^−1^), and $\delta_{s}$ C–H (1,080 cm^−1^). Figure 2B compares SERS (Au/MBA) with GERS (graphene/AuMBA). The SERS established a higher enhancement compared with the GERS phenomenon. The vibrational peaks were redshifted with the inclusion of the substrates. Although the graphene substrate solves the long-pending issue with irreproducibility of the metallic substrate in SERS, the low signal driven by charge transfer negates its application. Experimental techniques that combine graphene with metallic substrates have alleviated the issue of low enhancement. Figure 2C shows a comparison of Au/MBA (SERS) with a SERS + GERS coupled system. However, the simulated spectrum maintained a low intensity compared with Au/MBA SERS. The distance between the 4-MBA and the substrates possibly hindered the formation of hot spots and yielded a low intensity. To further investigate the relationship between GERS and SERS, experimental Raman data shown in Figure 3 depict the expected trend. The experimental SERS and GERS revealed an enhancement with the inclusion of the substrates. A clear trend is shown by the amplified Figure 3 insert of the C–C band. The SERS (Au/MBA) and Au/GERS both showed prominent enhancement emanating from the EM and CM. The subsequent section will detail the SERS/GERS mechanisms.

**FIGURE 4:**
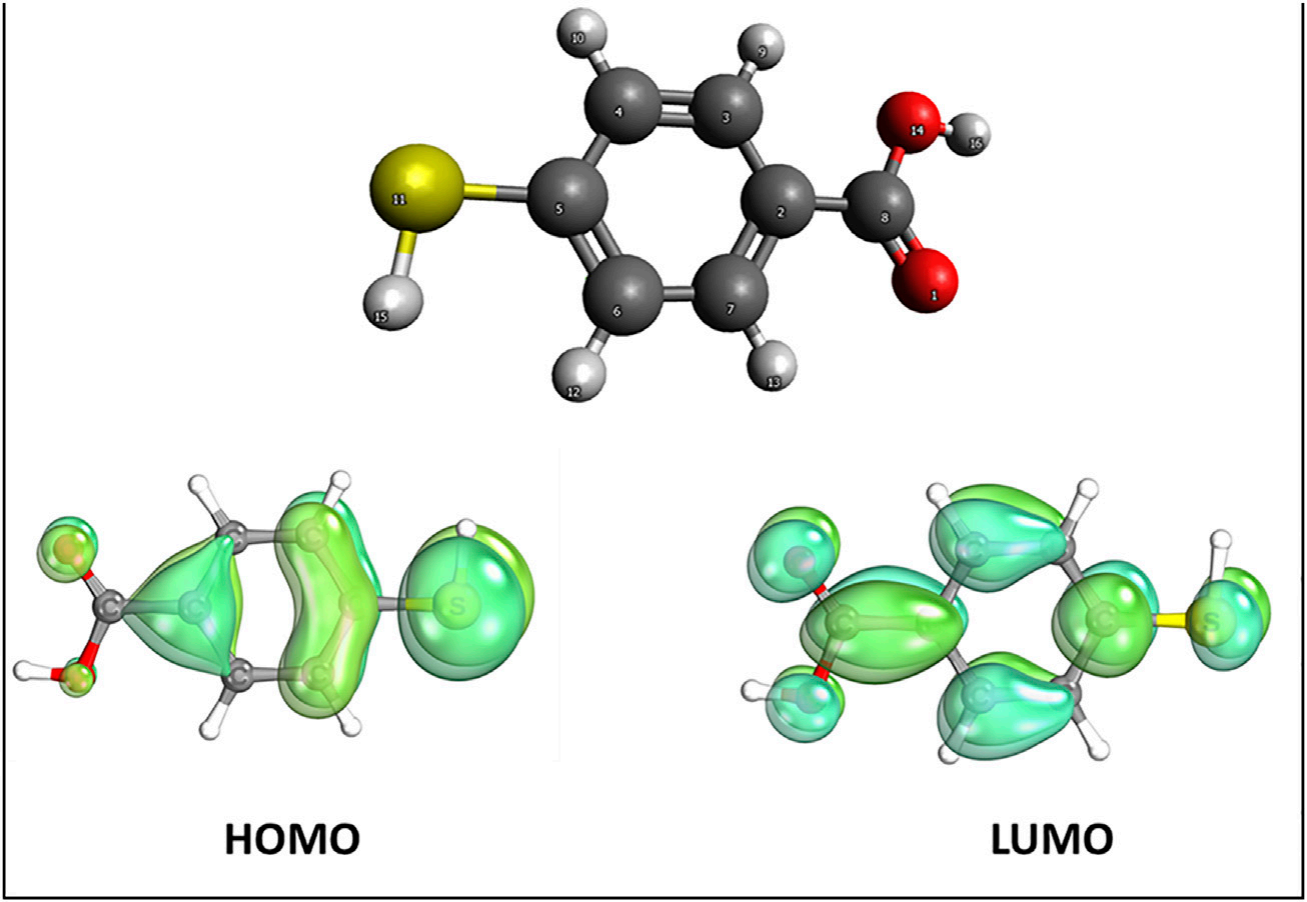
4-MBA molecular orbital plot.

### SERS/GERS mechanism

Frontier molecular orbitals (FMOs), i.e., the highest occupied molecular orbitals (HOMO) and lowest-lying unoccupied molecular orbitals (LUMO) determine the interaction of a molecule with other species (Demircioğlu et al., 2015). The HOMO, the outermost occupied orbital, is an electron-rich donor, while the LUMO, the innermost vacant orbital, is an electron acceptor (Demircioğlu et al., 2015). The MBA moiety HOMO and LUMO plots depict the thiol end as a better donor, electron-rich center compared with the carbonyl terminal. However, electron vacant orbitals are available on both terminals. Both terminals can accept electrons. The saturation of the HOMO on the thiol terminal explains the likelihood of the moiety interacting via the thiol end. Natural population analysis charges (NPA) and Mulliken charges are listed in Table 1. Although the NPA and Mulliken charges have different values, the charges are similar except for S, which carries a negative charge in Mulliken and a positive one in NPA. Also, the Wiberg–Mayer bond (MBO) indices confirmed covalent bonding in the respective bonds of the MBA with indices ranging from 0.7165 to 1.1283.

**TABLE 1:** 4-MBA Mulliken and natural population analysis (NPA) charges.

Atom	Mulliken charges	NPA charges
O1	−0.36568	−0.6036484509
C2	0.06817	−0.1969322834
C3	−0.20949	−0.1596222238
C4	−0.16158	−0.2435426919
C5	0.11176	−0.1530800157
C6	−0.19284	−0.2420717595
C7	−0.20511	−0.1553644705
C8	0.30486	0.7022508595
H9	0.15895	0.2442019043
H10	0.15659	0.2392696376
S11	−0.15825	0.0517322130
H12	0.15945	0.2389247867
H13	0.14914	0.2435293624
O14	−0.36639	−0.6480442342
H15	0.19400	0.1699708566
H16	0.35642	0.5124265091

**FIGURE 5:**
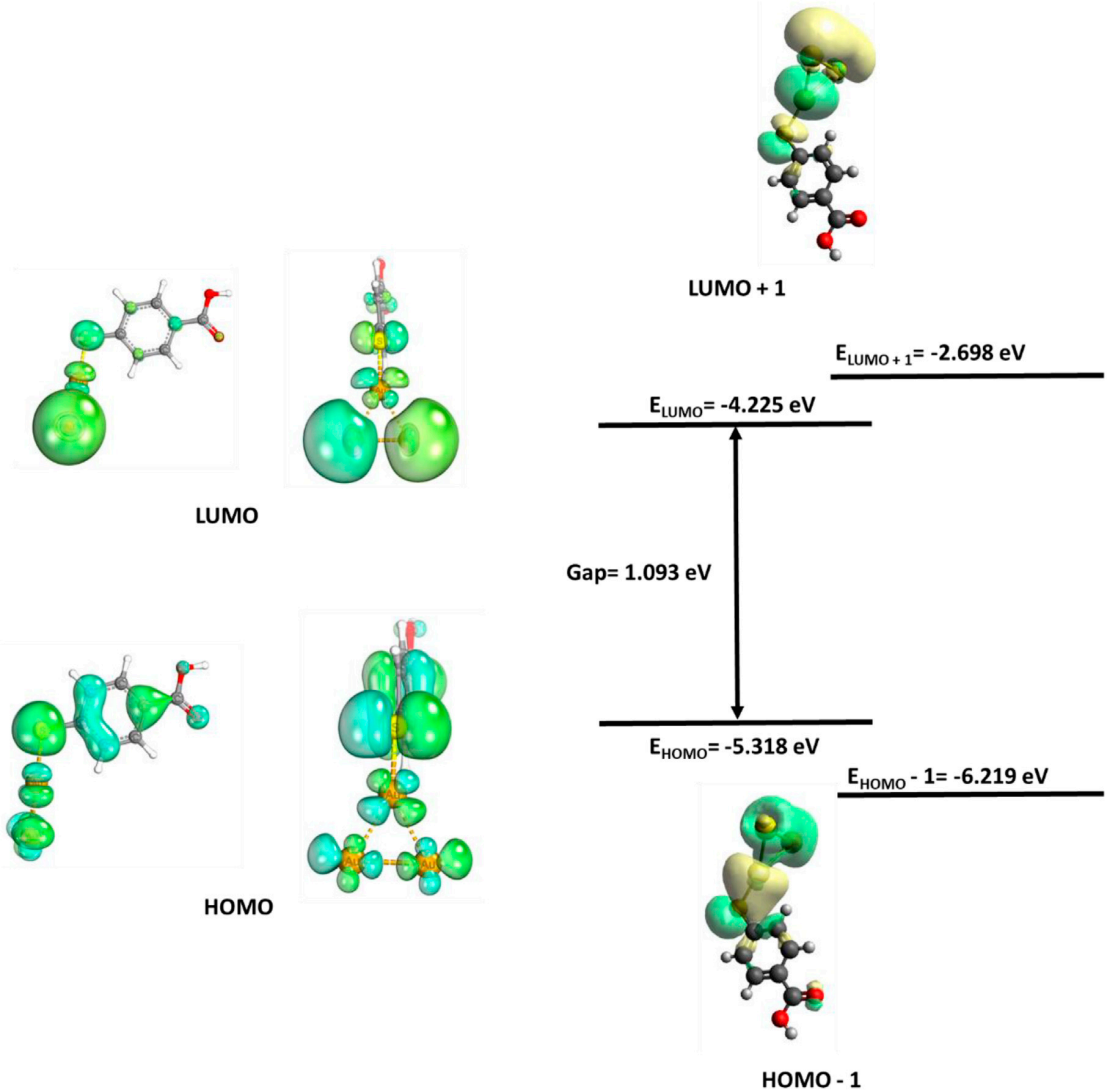
Au/MBA molecular orbitals.

**FIGURE 6:**
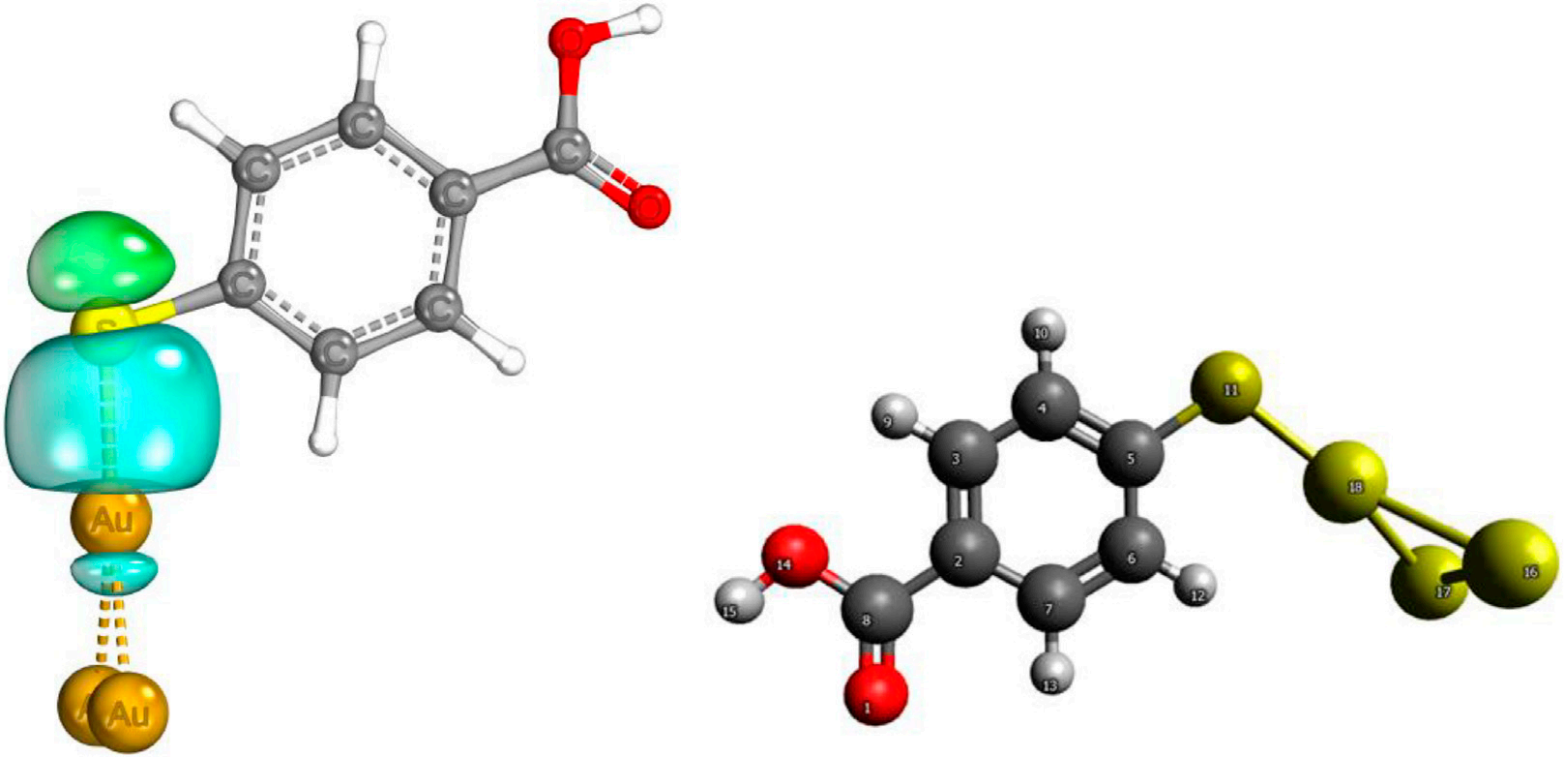
Au–S localized orbital.

**FIGURE 7:**
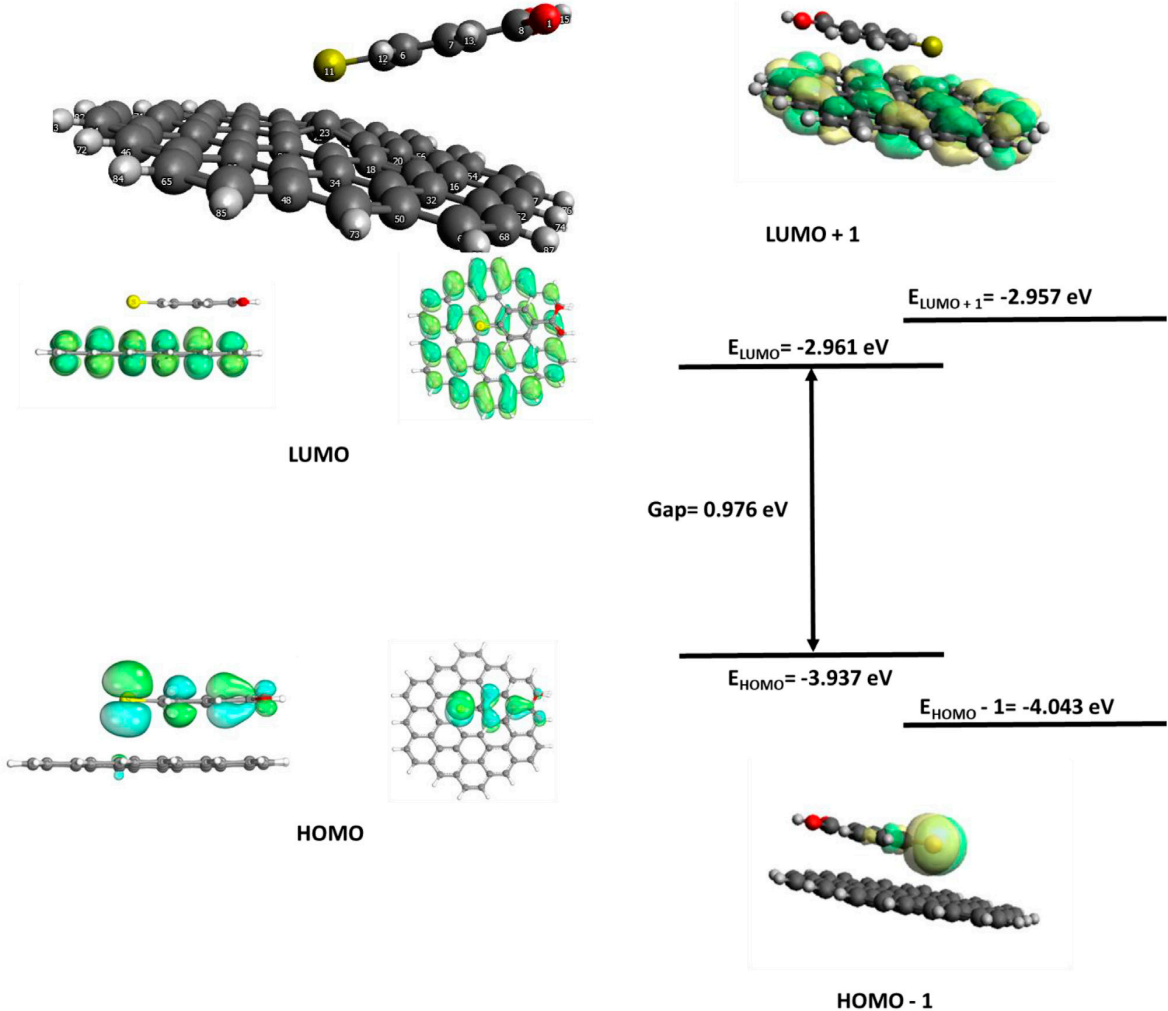
Graphene/MBA frontier molecular orbitals (FMOs).

**FIGURE 8:**
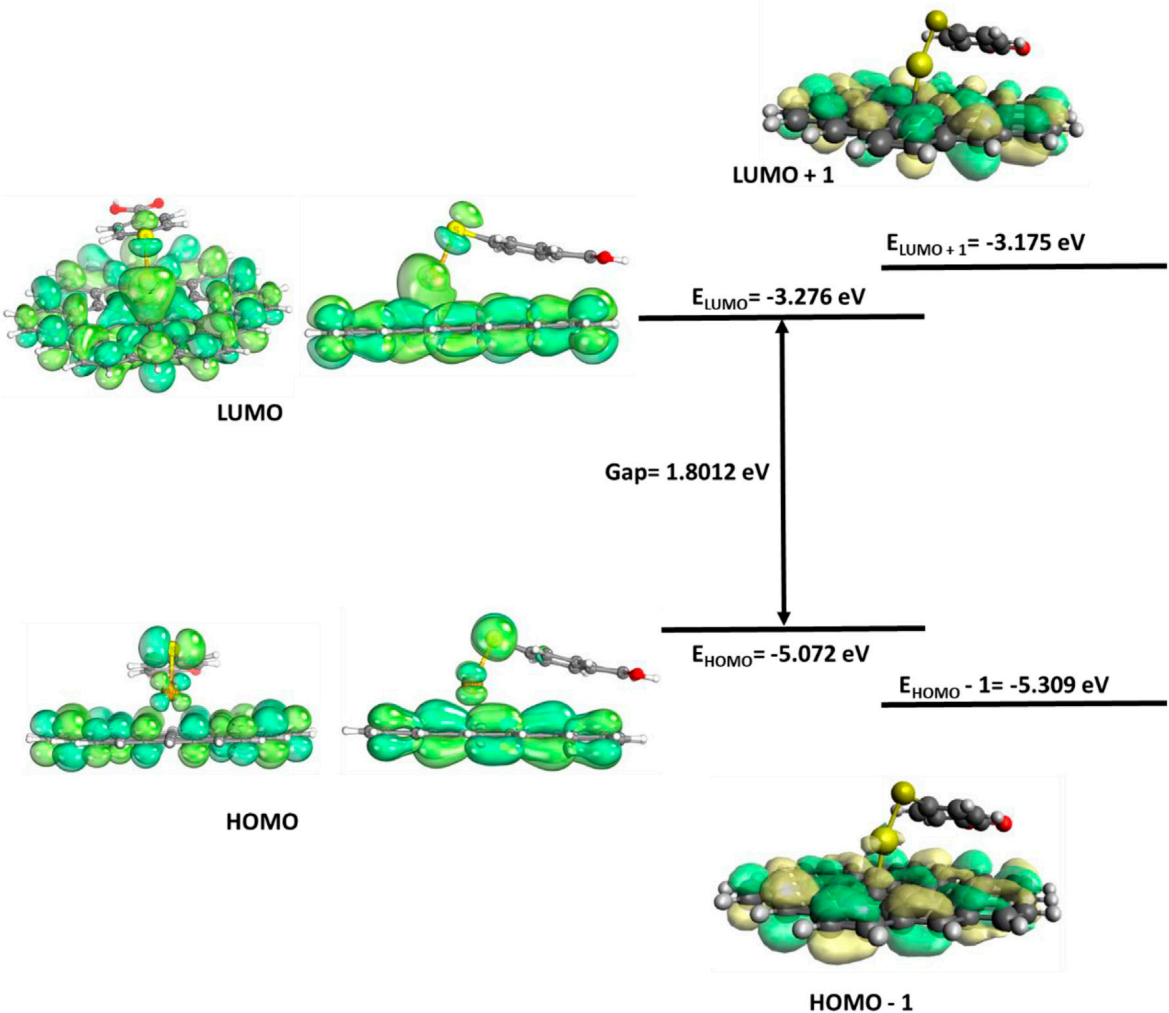
Au/GERS FMO plot.

To study the contribution of EM, charge transfer to the SERS and GERS signal FMOs were calculated and are shown in Figures 5–8. Figures 5, 7, and 8 depict HOMO, LUMO, HOMO +1, LUMO −1, and their energy eigenvalues. The HOMO–LUMO energies explain the charge transfer and insight into various reactivity such as ionization, electron affinity, hardness, softness, and chemical potential. Figure 5 depicts the HOMO and LUMO plots of Au/MBA SERS with a bandgap of 1.093 eV. The enhancement in Figure 5 is expected to be EM dominated. HOMO loops are concentrated on the 4-MBA, while LUMO is on the Au plasmonic substrate. In agreement with pristine 4-MBA HOMO, sulfur is a donor to the Au substrate acceptor. The donor–acceptor relationship between S–Au atoms attest to a charge transfer mechanism and is further confirmed by the localized orbital. The S and Au18 localized orbital depicted by Figure 6 corroborates the electron sharing between the two atoms with orbital energy of −0.425617 U. Analysis of the MBO indices of the Au–S bond (0.56332) endorses bond formation between the two; however, it is not strong enough to be claimed as covalent.

Partial charges and bond orbitals (IBOs) represent SCF wave functions and are based on intrinsic atomic orbital (IAO) charges in lieu of the Mulliken charges used for NPAs. The IBOs reflect classical boding concepts between atoms (Metz et al., 2000). The IBOs show S–Au σ localized bonding constituted by a lone pair (Figure 6). The IAO charges of the Au/MBA cluster are shown in Table 2 and, when compared with Mulliken and NPAs for pristine 4-MBA, show a decrease in charge with Au interaction. However, this bonding is not strong to be classified as covalent characterized by an MBO value of 0.56. On the contrary, the sensitivity of the MBO indexes to the choice of the basis set cannot be ignored and could have contributed to a lower index, thereby, discrediting a strong covalent bond. In a previous study, the use of 6-311G** basis set increased the MBO values to 1 (Peterson et al., 2003). The Au/MBA enhancement emanates from both EM and CM, with EM dominating the process.

**TABLE 2:** Au/MBA calculated charges.

Total charge composition						
CEN	ATOM	S	*p*	d	Electrons - > P. charge	
1	0	3.66906	4.81206		8.48111	−0.48111
2	C	2.93117	3.14486		6.07602	−0.07602
3	C	2.96795	3.15993		6.12787	−0.12787
4	C	2.96708	3.19055		6.15763	−0.15763
5	C	2.98530	3.09717		6.08248	−0.08248
6	C	2.96816	3.18696		6.15512	−0.15512
7	C	2.96834	3.15322		6.12155	−0.12155
8	C	2.87606	2.65433		5.53038	0.46962
9	H	0.83444			0.83444	0.16556
10	H	0.84073			0.84073	0.15927
11	5	5.77279	10.46979		16.24258	−0.24258
12	H	0.84848			0.84848	0.15152
13	H	0.83608			0.83608	0.16392
14	0	3.61056	4.83881		8.44937	−0.44937
15	H	0.61757			0.61757	0.38243
16	Au	3.00667	5.99799	9.89989	18.90455	0.09545
17	Au	3.00599	5.99800	9.90046	18.90445	0.09555
18	Au	3.06752	5.99899	9.72307	18.78958	0.21042

The GERS molecular orbitals are shown in Figure 7. The 4-MBA is physisorbed on the GERS surface. The optimized ground state of the GERS substrate, graphene, and a 4-MBA SERS tag, 4-MBA, shown in Figure 7 depicts a lack of covalent interaction between the two moieties. However, atom 23 was dislodged from the graphene planes. The 2-D graphene SERS substrate is documented for inducing the CM, i.e., prominent charge transfer. The graphene/4-MBA FMOs in Figure 7 enact the CM with the graphene substrate as an acceptor, while the 4-MBA is a donor. However, CM only contributes a smaller percentage of enhancement compared with EM, evidenced by the Raman spectra (Figure 2C).

**FIGURE 9:**
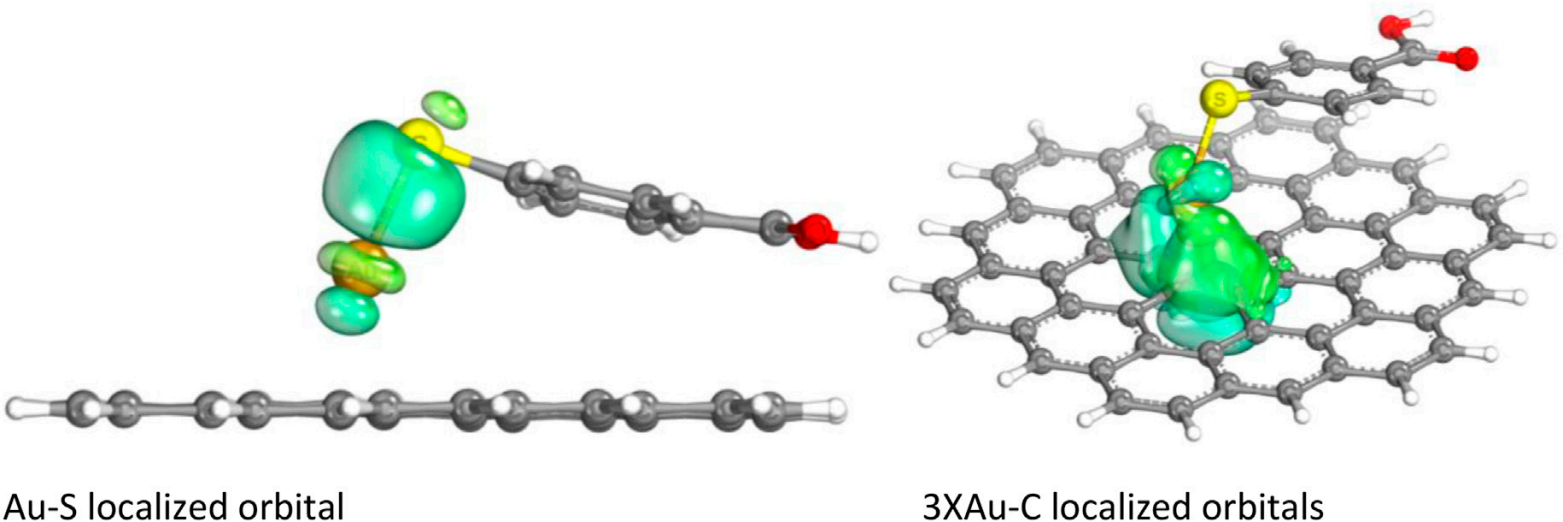
Au/GERS localized orbitals.

The SERS/GERS system (Au/GERS) HOMO–LUMO plots show donor centers on the graphene carbons and MBA sulfur atoms. The plasmonic Au metallic SERS substrate has electron-vacant orbitals with some of the graphene C atoms. Charge transfer emanates from S donor–Au acceptor and C donor–Au acceptor relations. Electron sharing in these three bonds is further corroborated by the localized orbitals in Figure 9. Figure 9 shows four sets of localized orbitals between S–Au and Au with three of the graphene carbons. This observation surmises an EM and CM-driven enhancement of the SERS and GERS system. On the contrary, the Raman spectrum (Figure 2C only showed an average enhancement, while experimental studies (Figure 3) commensurate both mechanisms. Literature has also evidenced better enhancements on the synergy of SERS and GERS. Hence, it is cogent to explore the SERS and GERS approaches, which alleviate metallic substrate issues and evoke both EM and CM.

**SKETCH 1:**
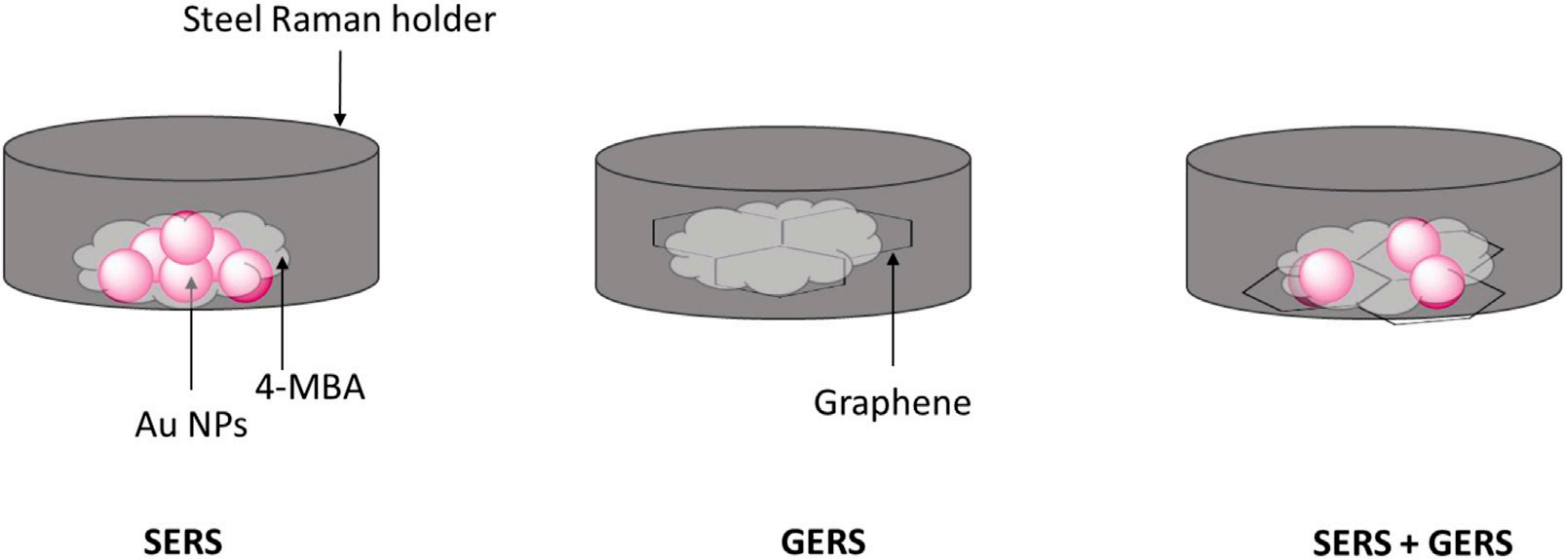
SERS, GERS, and SERS combined with GERS schematic representations.

The LUMO and HOMO loops of Au/GERS show a similar distribution of both donors and acceptor centers mainly located on the C–C of the graphene GERS substrate. The difference is observed with the Au and S atom, with S inheriting an electron-rich donor status, while Au is an electron-deficient acceptor.

The Au/GERS cluster had four localized orbitals: Au–S and Au and 3 C atoms of the graphene substrate. The localized orbitals confirm the sharing of electrons between the Au–S and Au–C. The Au center is electron rich and donates electrons to the S and carbons. This also proves charge transfer between the atoms.

## Conclusion

Based on the foregoing results, it is clear that both plasmonic metal SERS and graphene-enhanced Raman spectroscopy (GERS) hold promise for the development of functional SERS for the diagnosis of diseases. GERS is more promising in respect to reproducibility. However, low enhancement remains a challenge. By way of introducing plasmonic metal to a graphene layer, an atom-thick flat substrate (2-D-layered structure), SERS was successfully developed. Thus, this was done through the assembly of a SERS/GERS system leading to Au SERS substrate having vacant orbitals with some graphene carbon atoms. It was elucidated that the enhancement capabilities of the SERS/GERS system was amplified partially through CM with EM showing significant dominance. Although still outperformed by Au/MBA, Au/GERS substrate shows a unique application of graphene, and it is anticipated to benefit both the deeper understanding of the SERS effect itself and various practical applications of SERS.

## Data Availability

The raw data supporting the conclusion of this article will be made available by the authors, without undue reservation.
